# A proposal for using benefit-risk methods to improve the prominence of adverse event results when reporting trials

**DOI:** 10.1186/s13063-024-08228-0

**Published:** 2024-06-22

**Authors:** Nikki Totton, Ed Waddingham, Ruth Owen, Steven Julious, Dyfrig Hughes, Jonathan Cook

**Affiliations:** 1 Sheffield Centre for Health and Related Research, School of Medicine and Population Health, University of Sheffield, Regent Court, 30 Regent Street, Sheffield, S1 4DA UK; 2 Imperial Clinical Trials Unit, School of Public Health, Imperial College London, London, UK; 3 Department of Medical Statistics, London School of Hygiene and Tropical Medicine, London, UK; 4 Centre for Health Economics and Medicines Evaluation, Bangor University, Bangor, UK; 5 Centre for Statistics in Medicine, Nuffield Department of Orthopaedics, Rheumatology and Musculoskeletal Sciences, University of Oxford, Oxford, UK

**Keywords:** Adverse events, Harms, RCT reporting, Benefit-risk

## Abstract

Adverse events suffer from poor reporting within randomised controlled trials, despite them being crucial to the evaluation of a treatment. A recent update to the CONSORT harms checklist aims to improve reporting by providing structure and consistency to the information presented. We propose an extension wherein harms would be reported in conjunction with effectiveness outcome(s) rather than in silo to provide a more complete picture of the evidence acquired within a trial. Benefit-risk methods are designed to simultaneously consider both benefits and risks, and therefore, we believe these methods could be implemented to improve the prominence of adverse events when reporting trials. The aim of this article is to use case studies to demonstrate the practical utility of benefit-risk methods to present adverse events results alongside effectiveness results. Two randomised controlled trials have been selected as case studies, the Option-DM trial and the SANAD II trial. Using a previous review, a shortlist of 17 benefit-risk methods which could potentially be used for reporting RCTs was created. From this shortlist, three benefit-risk methods are applied across the two case studies. We selected these methods for their usefulness to achieve the aim of this paper and which are commonly used in the literature. The methods selected were the Benefit-Risk Action Team (BRAT) Framework, net clinical benefit (NCB), and the Outcome Measures in Rheumatology (OMERACT) 3 × 3 table. Results using the benefit-risk method added further context and detail to the clinical summaries made from the trials. In the case of the SANAD II trial, the clinicians concluded that despite the primary outcome being improved by the treatment, the increase in adverse events negated the improvement and the treatment was therefore not recommended. The benefit-risk methods applied to this case study outlined the data that this decision was based on in a clear and transparent way. Using benefit-risk methods to report the results of trials can increase the prominence of adverse event results by presenting them alongside the primary efficacy/effectiveness outcomes. This ensures that all the factors which would be used to determine whether a treatment would be recommended are transparent to the reader.

## Background

Adverse events are routinely collected within all randomised controlled trials (RCTs) to provide an important assessment of harms [1]. Despite their importance in supporting a claim for an acceptable harms profile, they are not usually considered within the main design or primary analysis of the RCT which tends to focus on the main effectiveness outcome [2]. As a consequence, it has been reported that analysis of adverse events is lacking in power [3] and is not currently sufficient or being used to the data’s full potential [4]. Furthermore, Phillips and Cornelius [5] argue that the pairing of better analysis with effective reporting is needed to allow the successful assessment of harms within RCTs.

Currently, the reporting of adverse events is deemed to be inadequate [3] and suboptimal [6] leading to difficulties in understanding what has been collected and how it has been assessed [7]. The reporting of harms has aimed to be improved by the introduction of the CONSORT checklist for harms, originally created in 2004 [8] and updated in 2022 [9]. A review suggested that the original CONSORT harms checklist did improve reporting; however, the improvement was limited, and there was continued room for progression in this area [10].

It is not unusual that the evaluation of treatment is influenced by the adverse event results. For example, an RCT assessing diet treatments for children with drug-resistant epilepsy found that a low glycaemic index diet was inferior to a ketogenic diet on efficacy [11]. However, the treatment-related adverse events were significantly lower in the low glycaemic index group meaning that the overall conclusion by the authors was that treatment decisions should be made individually. Therefore, it is not just an improvement of the reporting of harms in silo that is being suggested, but also in conjunction with the reporting of the effectiveness outcomes. This is to ensure the totality of evidence generated within any given RCT is able to be appropriately considered [3]. This reflects the information required by both clinicians and patients to make suitable treatment decisions [6]. This also mirrors the regulatory process to gain approval for a new treatment which requires the submission of data on all important outcomes including both effectiveness and attributable harms often using a benefit-risk framework [12].

In a 2017 review [10], it was found that 58% (579/996) of RCTs reported a balanced discussion of benefits and harms (as per item 10 in the CONSORT extension for harms), again demonstrating the importance that the data for both of these aspects is not only available but also clearly reported. The possibility to complete a benefit-risk assessment is also mentioned by many researchers when discussing the analysis and/or reporting of harms [1, 3, 13]. The recent update to the CONSORT harms has integrated the harms checklist into the main CONSORT checklist instead of considering it as a standalone. This is said to reflect ‘the need for balance in reporting both harm and benefit’ [5]. There is the potential for benefit-risk methods to be useful in this context to ensure this requirement is satisfied.

Benefit-risk (B-R) methods are a group of methods [14–16] that allow both the benefits (efficacy/effectiveness) and risks (adverse events/harms) to be simultaneously and systematically considered. The purpose of this is to support an overall judgement on the appropriateness of the treatment.

It has been suggested that the lack of a more comprehensive analysis of adverse events data is due to the space constraints within an article [4]. The use of B-R methods could be a solution to this issue as they can efficiently summarise the results in a structured manner. Work previously completed to evaluate the potential to implement B-R methods in publicly funded RCTs highlighted the benefits of these methods as improving the transparency and consistency of RCT reporting [17]. Additionally, the use of a B-R style summary table was recommended for reporting the findings of an RCT [17]. The rationale being this will ensure the clear presentation of all important aspects of the treatment which will enable suitable treatment decisions to be made based on the information provided.

Examples exist where B-R methods have been used to summarise the effectiveness against the harms using data from an RCT. These are in clinical areas such as cardiology [18], cancer [19] and multiple sclerosis [20]. Given the use of these methods to successfully summarise the benefits and risks found from RCT data and their use for summarising data to achieve regulatory approval, it could be argued that there should be more widespread adoption of these methods when reporting the results of a RCT.

The aim of this article is to illustrate, using worked examples, the use of B-R methods to present adverse event results alongside effectiveness results. The intention is to help support the application of selected methods in the reporting of RCTs by illustrating their practical utility.

## Methods

### Benefit-risk method selection

The B-R methods represented in the case studies below have been taken from a previous rapid systematic review which outlined available B-R methods for use in publicly funded RCTs [17]. From the initial list of 96 methods, those deemed not relevant (*n* = 29), not appropriate due to only considering 1 outcome (*n* = 5) or requiring extra data to that typically collected in an RCT (*n* = 45) are not taken forward. The remaining 17 methods which could be potentially useful in this scenario spanned across different method types (as defined by the PROTECT group [14]) including descriptive or quantitative frameworks, trade-off metrics and visual methods (Table 1). This list is not meant to be exhaustive, but rather a shortlist of potential methods as a starting point for those looking to improve the reporting of adverse events.

**Table 1:** Shortlist of appropriate benefit-risk methods for use when presenting adverse event data in RCTs

Method	Method type
Benefit-Risk Assessment for Non-prescription Drugs (BRAND)[22]	Descriptive framework
Consortium On Benefit-Risk Assessment (COBRA) [23]	Descriptive framework
Summary table	Descriptive framework
Benefit-Risk Action Team (BRAT) Framework [24]	Descriptive framework
FDA Benefit-Risk Framework (BRF) [25]	Descriptive framework
PrOACT-URL [20]	Descriptive framework
Net clinical benefit (NCB) [26]	Quantitative framework
Quantitative Framework for Risk and Benefit Assessment (QFRBA) [16]	Quantitative framework
Beckmann [14]	Trade-off metric indices
Outcome Measures in Rheumatology (OMERACT) 3 × 3 table [27]	Trade-off metric indices
Tables	Visual
Bar charts	Visual
Benefit-Harm Charts [28]	Visual
Box plots	Visual
Dot chart	Visual
Forest plot	Visual
Tree diagram	Visual

Many of the visual methods are not specific to benefit-risk methods, and as comprehensive research into the optimum visual presentation of adverse events is available [21], these methods will not be considered in further detail. One method was selected for illustration purposes from each of the remaining method type groups. The authors selected the methods based on their perceived usefulness to illustrate improved adverse event reporting and well as those that are commonly used within the literature. Methods selected were the Benefit-Risk Action Team (BRAT) Framework, net clinical benefit (NCB) and the Outcome Measures in Rheumatology (OMERACT) 3 × 3 table.

The BRAT framework will be applied to both case studies whereas NCB will be applied to case study 2 and OMERACT table to case study 1 only. This is due to the primary outcome data type which was continuous for case study 1 and binary for case study 2. The OMERACT method requires categorisation into three to represent the deemed strength of the benefit which was possible on the primary outcome due to the summaries provided by the case study 1 results. This categorisation should be straightforward for the presence of certain adverse events but will require the categorisation of any outcomes such as pain level into categories that is considered none/minor, major and (near) death. However, dichotomisation of this outcome would have required potentially further clinical knowledge. As NCB is related to the probability of an event occurring this is directly applicable for the presence of adverse events and especially easy when the primary outcome is also binary as in case study 2. To note here, a potential solution to dichotomise continuous outcomes would be to use a distributional approach as recommended for continuous adverse events [29].

### Benefit-risk methods description

#### Benefit-Risk Action Team (BRAT) Framework

The Benefit-Risk Action Team (BRAT) Framework was first reported in 2011 to improve the expert judgement of clinical decision-making by being more ‘transparent, rational and defensible’ [24]. This framework was created to provide a set of guiding principles that were suitable for use by all stakeholders to complete a benefit-risk assessment and concluded with a six-step process as follows:

1. Defining the decision context
2. Identifying the benefit and risk outcomes
3. Identifying data sources
4. Customising the framework
5. Assessing the relative importance of the outcomes
6. Displaying and interpreting the key benefit-risk metrics

As we can assume steps 1–5 are completed within the design and conduct of an RCT, this paper will focus on step 6—displaying the results; however, the value tree in step 2 will be presented for completeness.

For reporting, the BRAT framework recommends the use of a key benefit-risk summary table which they state has been ‘designed to allow users to readily grasp the major issues underlying a benefit-risk assessment’ [24]. This table provides both the key benefits and risks separately and alongside each other which allows direct comparison but no formal calculation of trade-off between outcomes is completed. Benefits in this case are defined as favourable events and risks are any unfavourable events potentially associated with the treatments. The structure of the table, at a minimum, shows the event rates for both treatment groups, the difference (on the same scale for each outcome if possible) and the variability of this difference. There is the ability to add additional information such as stakeholder preferences if it is available and useful.

The creators provided a worked example of the BRAT framework [30] which gave further details and key considerations. In this example, all of the outcomes are binary and therefore can all be considered on a risk difference scale (adjusted to per x patients for comparability). However, continuous outcomes are common within RCTs. The authors of the BRAT framework originally stated continuous measures can be included if they can be dichotomised [30] but due to the loss of information with this [31], we believe these can be included without dichotomisation by using a standardised effect size measure for comparability and this has been implemented within the case studies. A pilot programme using the BRAT framework in practice was completed in 2012 and found it to be effective and flexible to different circumstances with the key benefit-risk summary table to be a particularly attractive element [32].

#### Net clinical benefit (NCB)

The net clinical benefit (NCB) framework was developed in the context of individual patient decision-making [33] but can also be used to assess the average benefit-risk balance of treatments among a trial population. It is a quantitative framework which addresses the need to weigh up all the benefits and risks of treatment simultaneously. When applied to a particular treatment, the method generates a metric, the expected net clinical benefit, calculated as the expected overall benefit of treatment less the expected overall harm.

Within the NCB framework, benefits and harms must be characterised in terms of probabilities, this is straightforward to do for binary events (see SANAD II case study) whilst for non-binary events, additional calculations are required. Furthermore, in order to integrate the benefits and harms into a single metric, they should be expressed on a comparable scale; in general, this will require weighting each benefit and harm in the NCB calculation in proportion to its impact, using elicited utilities or quality-of-life data, for example. The general NCB equation then becomes:

$$\text{Expected NCB }= \sum_{i\epsilon B}{w}_{i}{p}_{i}-\sum_{i\epsilon H}{w}_{i}{p}_{i}$$

where *B* is the set of benefits,* H* is the set of harms, ${w}_{i}$ is the weight associated with outcome *i* and ${p}_{i}$ is its probability of occurrence.

To compare treatments in terms of their overall benefit-risk profile, the difference in expected NCB between each treatment should be calculated—treatments with higher expected NCB have a better benefit-risk balance on average.

In simple cases with small numbers of benefits and harms and/or where all events are of similar impact, it may be possible to determine which treatment has a higher NCB without the use of explicit weightings, but in such cases, caution should always be taken to ensure the conclusions are robust.

#### Outcome Measures in Rheumatology (OMERACT) 3 × 3 table

In rheumatology RCTs, researchers realised the need for a simple method that could consider both the benefits and risks [27]. This method builds on the principle of the threes method [34] whilst remaining more straightforward than some of the more complex benefit-risk methods. The method simply categorises patients into whether their overall benefit was none/minimal, major or (near) remission. The same is then considered for the patient’s overall harm being none/minimal, major, or (near) death. A 3 × 3 table is created using these two categories to show the percentage of patients in each subset. The table is colour-coded to represent the benefit/risk with black indicating no benefit with high risk and white showing high benefit with no risk and a mixture of red, orange and yellow used for the cells in between. A separate table is created for each treatment which allows direct visual comparison, but there is no formal numerical comparison between treatment options.

The strength of this method is in the trade-off of outcomes at the patient level, instead of the population level, which is recommended for benefit-risk assessments [35, 36]. The authors recognised the drawbacks of simplification that occurs when only three categories are used but highlight that it avoids a complex weighting process whilst still being able to group similarly important outcomes together. At a glance, it allows the reader to see whether patients are benefiting with/without harms, and therefore, a reflection of the overall benefit-risk summary of the treatment. Although criticism states that as this is over two tables it can be hard to directly compare [26].

### Case studies

#### Option-DM

Option-DM [37] was an RCT (*n* = 140) for patients with diabetic peripheral neuropathic pain as four different treatment drugs were recommended so a direct comparison of these was required. This was a crossover trial which randomised equally to six different treatment pathways. For simplicity, the data from just two of the treatment pathways (monotherapy of amitriptyline and combination therapy of pregabaline followed by amitriptyline) will be used for demonstration purposes. The primary outcome was the 7-day average daily pain at the end of each pathway, although this is a continuous outcome the team have provided different dichotomisation options of a 30% pain reduction, a 50% pain reduction or a pain score of less than 3 (low pain).

The RCT showed similar efficacy across the treatment pathways but an improvement in change in pain score for those on combination therapies compared with monotherapies (mean change 1.0 [SD = 1.3] vs 0.2 [SD = 1.5]). Secondary patient-reported outcomes however were only presented as continuous outcomes. Adverse events of dizziness, nausea and dry mouth were key findings from the RCT as described in the abstract and in total 16 different adverse events were reported tabularly.

##### Current display of adverse events

In the main publication [37], reporting of adverse events was restricted to those that were treatment-emergent and occurring in more than 5% of patients. These are summarised in tabular form as the number (%) of patients experiencing each event, together with *P*-values from global chi-squared tests comparing the rates of adverse events between therapies. Table 2 shows the summaries of the number of adverse events for the two treatments of interest, monotherapy of amitriptyline and combination therapy of pregabaline followed by amitriptyline.

**Table 2:** Summary of the number of adverse events in the Option-DM RCT, reproduced from the results paper [37]

**Adverse event**	**Monotherapy amitriptyline (*n*=104)**	**Combination therapy pregabaline-amitriptyline (*n*=107)**
Fatigue	18 (17%)	22 (21%)
Dry mouth	22 (21%)	18 (17%)
Dizziness	8 (8%)	26 (24%)
Sedation	19 (18%)	15 (14%)
Diarrhoea	8 (8%)	9 (8%)
Nausea	4 (4%)	7 (7%)
Oedema	2 (2%)	17 (16%)
Constipation	9 (9%)	8 (7%)
Headaches	8 (8%)	8 (7%)
Fall	3 (3%)	10 (9%)
Excessive sweating	7 (7%)	6 (6%)
Vomiting	5 (5%)	8 (7%)
Insomnia	3 (3%)	7 (7%)
Abdominal cramping	4 (4%)	4 (4%)
Ataxia	1 (1%)	8 (7%)
Inability to concentrate	4 (4%)	6 (6%)

The more detailed study report [38] summarises a complete list of adverse events, both as the number of events and as the number of patients experiencing an event. In addition, adverse events are presented by treatment phase (< 6 weeks or > 6 weeks), as a subset of those that are moderate or severe and as a subset of those that are moderate or severe and related to study treatment. Bar charts showing the mean number of days affected by each adverse event per 1000 days follow-up are reported, together with tabular summaries of average weeks affected per person and days affected per 1000 days follow-up. The number of serious adverse events and the number of patients experiencing serious adverse events are also summarised by treatment pathway (any serious adverse event and the eight most common serious adverse events), although few occurred in the study (*n* = 31).

##### BRAT value tree and table

**Fig. 1:**
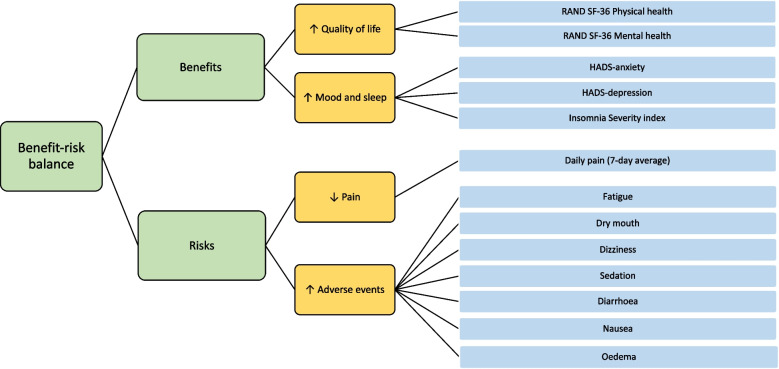
BRAT value tree for the Option-DM RCT

The value tree (Fig. 1) clearly outlines the key benefits (favourable outcomes) and risks (unfavourable outcomes) from the RCT which will go into the overall benefit-risk assessment of the treatments. The favourable outcomes within the RCT are quality of life as well as mood and sleep which are measured using a range of patient-reported outcome measures. The primary outcome of a 7-day average of daily pain is included as an unfavourable outcome in the value tree, it is assumed this will be improved by the treatment and pain will be reduced. Additionally, the most common adverse events were included as risks. The outcomes identified in the value tree are also used within the BRAT summary table (Table 3) which shows the results of both treatments and the treatment difference (with confidence intervals).

**Table 3:** BRAT summary table for the Option-DM RCT

		**Outcome**	**Combination therapy pregabaline-amitriptyline (*n* = 107)**	**Monotherapy amitriptyline (*n* = 104)**	Standardised effect size (95% CI)*
**Benefits**	↑Quality of life	RAND SF-36 Physical health, *mean (SD)*	24.1 (13.1)	25.4 (12.5)	− 0.10 (− 0.37, 0.17)
		RAND SF-36 Mental health, *mean (SD)*	47.4 (12.3)	46.7 (13.0)	0.06 (− 0.21, 0.33)
	↑ Mood and sleep	HADS-anxiety, *mean (SD)*	7.0 (4.6)	7.5 (5.1)	− 0.10 (− 0.37, 0.17)
		HADS-depression, *mean (SD)*	7.2 (4.5)	7.4 (4.7)	− 0.04 (− 0.31, 0.23)
		Insomnia Severity index *mean (SD)*	12.1 (6.4)	11.8 (5.9)	0.05 (− 0.22, 0.32)
					**Odds ratio (95% CI)**
**Risks**	↓ Pain	30% reduction in daily pain, *n (%)*	68 (64%)	68 (65%)	0.92 (0.53, 1.62)
		50% reduction in daily pain, *n (%)*	47 (44%)	42 (40%)	1.16 (0.67, 2.00)
	↑Adverse events	Fatigue, *n (%)*	22 (21%)	18 (17%)	1.24 (0.62, 2.47)
		Dry mouth, *n (%)*	18 (17%)	22 (21%)	0.75 (0.38, 1.51)
		Dizziness, *n (%)*	26 (24%)	8 (8%)	3.85 (1.65, 8.97)
		Sedation, *n (%)*	15 (14%)	19 (18%)	0.73 (0.35, 1.53)
		Diarrhoea, *n (%)*	9 (8%)	8 (8%)	1.10 (0.41, 2.98)
		Nausea, *n (%)*	7 (7%)	4 (4%)	1.75 (0.50, 6.27)
		Oedema, *n (%)*	17 (16%)	2 (2%)	9.63 (2.17, 42.85)

Using this summary table, the reader can easily see that there is no discernible difference between the treatments on any of the benefit categories. However, a difference is apparent in some of the adverse events with two particularly large differences (dizziness and oedema) in favour of the monotherapy amitriptyline.

##### OMERACT 3 × 3 table

Application of the OMERACT 3 × 3 table required a decision of which outcomes represent none/minimal, major and near remission/death for the benefits and risks. The categories chosen based on the information contained in the RCT outputs are outlined below. For simplicity, only the primary outcome of reduced pain has been chosen for the benefit and categorised by a percentage improvement. However, this can be extended to include multiple outcomes as a composite using patient-level data.

The following are the benefits:

- (Near) remission: pain reduction of more than 50%
- Major: pain reduction of more than 30% but less than 50%
- None/minimal: pain reduction of less than 30%

The following are the risks:

- (Near) death: serious adverse event
- Major: moderate or severe treatment-related adverse events (as defined by the trial team)
- None/minimal: no adverse events or minor adverse events

As we do not have access to the patient-level data for the Option-DM RCT, three versions of the 3 × 3 table have been created (Tables 4 (a–c)) which all indicate different potential patterns that the data could show given the summary statistics we have access to. The purpose of completing three versions is to demonstrate the additional information that can be provided by using this method and how this could influence opinions of treatment. The total column has been taken from the data presented in the outputs and just the cell data created. Table 4 (a) represents a situation where there was no discernible relationship between the benefits and risks and the percentages are proportional to the total columns. Table 4 (b) shows a skew towards higher benefits with the treatment being related to also having higher risks. Finally, Table 4 (c) suggests two subsets of patients with a skew towards some with high benefits having lower risks and vice versa with those patients with higher risks seeing lower benefits. Clearly, this will create three very different conclusions to the treatment benefit-risk balance.

**Table 4:**
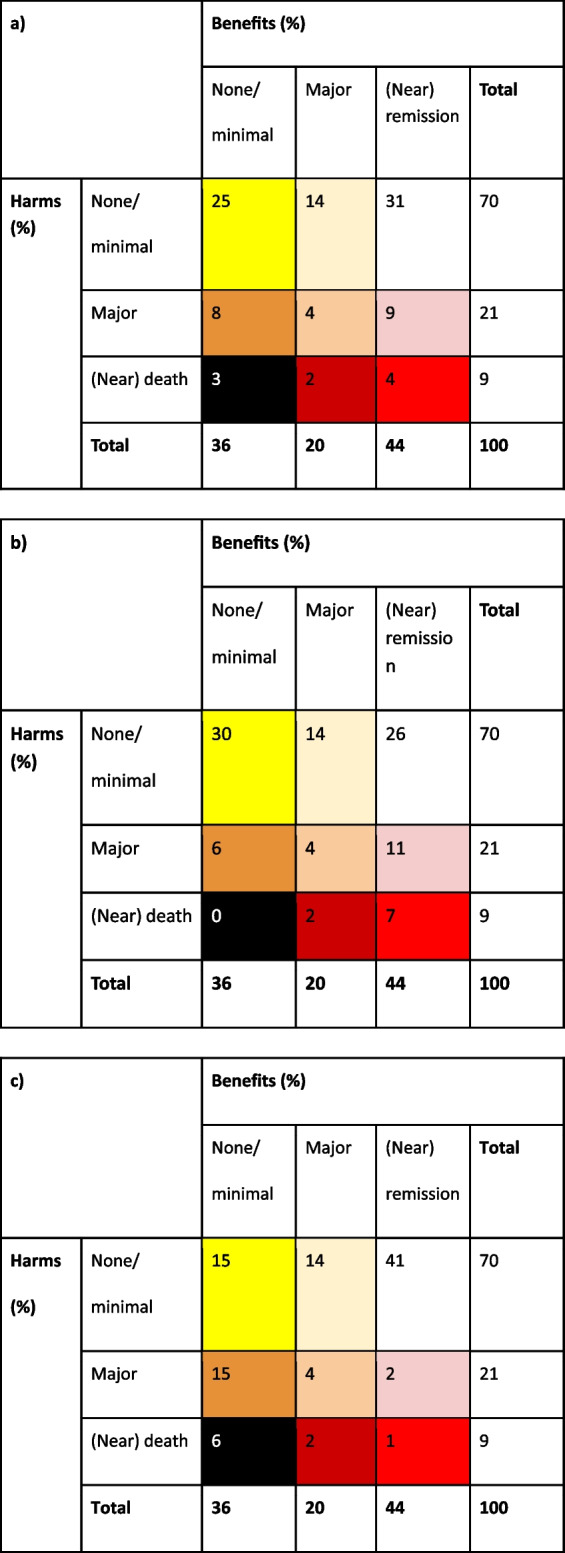
OMERACT 3 × 3 table for the OPTION-DM RCT for those on combination therapy pregabaline followed by amitriptyline (*n*=107) where the responses are (a) proportional, (b) skewed towards higher benefits related to higher risks and (c) skewed towards two groups of patients with high benefits/low risks and high risks/low benefits

#### SANAD II

The SANAD II study [39] was a non-inferiority RCT for patients with newly diagnosed focal epilepsy (*n* = 990). Patients were equally randomly allocated to receive either lamotrigine (control treatment), levetiracetam (treatment 1) or zonisamide (treatment 2), which are both licenced for use but longer-term effectiveness data were not available.

The primary outcome, assessed on a non-inferiority basis, was time to 12-month remission (non-inferiority margin hazard ratio (HR) of 1.329, 10% absolute difference). Levetiracetam was found not be non-inferior to lamotrigine (intention to treat analysis of time to 12-month remission HR = 1.18; 97.5% CI [0.95–1.47]). However, zonisamide was found to be non-inferior to lamotrigine (HR = 1.03; 97.5% CI [0.83–1.28]).

Analysis of adverse events showed that fewer participants with reactions for those in the lamotrigine arm (*n* = 108/328, 33%) than both the levetiracetam (*n* = 144/330, 44%) and zonisamide (*n* = 146/324, 45%) arms. Lamotrigine was superior in the cost-utility analysis and therefore, despite being clinically non-inferior, zonisamide was not recommended by the clinicians due to the increased adverse reactions identified and lamotrigine continues to be recommended as the first-line treatment. In this project for simplicity, we will only be reporting two of the treatments (lamotrigine and zonisamide).

##### Current display of adverse events

Within the results article for the SANAD II study [39], the number of adverse events and number and percentage of patients experiencing adverse events were reported by MedDRA system organ classification in the main publication as shown in Table 5. In the article's supplementary appendix, a more granular breakdown was given by MedDRA preferred term, and a separate table summarised only those adverse events classified as severe. A listing of all serious adverse reactions (SARs) was provided in the full study report [40] which included the seriousness, severity, expectedness, suspected relationship to the study drug, action taken and outcome. No formal statistical comparisons between treatment arms were performed.

**Table 5:** Summary of adverse reactions in the SANAD II RCT, reproduced from the results paper and HTA report [39, 40]

**Adverse reaction category**	**Adverse reaction count**		**Patients with at least one adverse reaction**	
	**Lamotrigine**	**Zonisamide**	**Lamotrigine (*n*=328)**	**Zonisamide (*n*=324)**
Psychiatric disorders	58	103	43 (13%)	73 (23%)
Nervous system disorders	88	85	53 (16%)	60 (19%)
General disorders and administration site conditions	23	44	17 (5%)	39 (12%)
Gastrointestinal disorders	30	35	25 (8%)	26 (8%)
Skin and subcutaneous tissue disorders	29	28	24 (7%)	21 (7%)
Investigations	6	16	6 (2%)	16 (5%)
Metabolism and nutrition disorders	4	17	3 (1%)	16 (5%)
Musculoskeletal and connective tissue disorders	5	8	5 (2%)	7 (2%)
Eye disorders	1	5	1 (< 1%)	5 (2%)
Renal and urinary disorders	1	6	1 (< 1%)	5 (2%)
Cardiac disorders	2	1	2 (1%)	1 (< 1%)
Respiratory, thoracic and mediastinal disorders	1	2	1 (< 1%)	2 (1%)
Injury, poisoning and procedural complications	2	0	2 (1%)	0 (0%)
Ear and labyrinth disorders	0	0	0 (0%)	0 (0%)
Endocrine disorders	0	0	0 (0%)	0 (0%)
Pregnancy, puerperium and perinatal conditions	0	1	0 (0%)	1 (< 1%)
Vascular disorders	1	0	1 (< 1%)	0 (0%)
**Total**	**251**	**351**	**108 (33%)**	**146 (45%)**

##### BRAT value tree and table

**Fig. 2:**
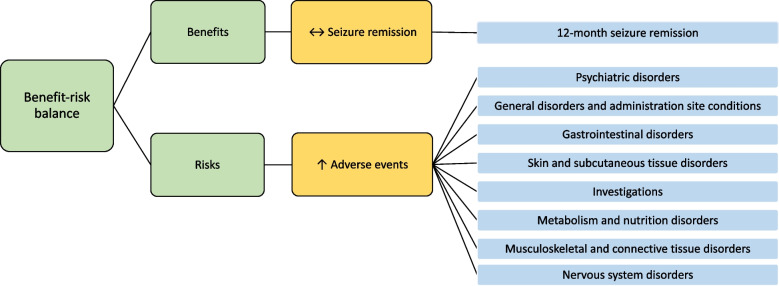
BRAT value tree for the SANAD II RCT

As this is a non-inferiority trial, the BRAT value tree (Fig. 2) may look slightly different as one of the outcomes, in this case seizure remission, is expected to be similar instead of better/worse. This is the main favourable outcome within this RCT and the adverse events are the main unfavourable outcomes.

The adverse events have been categorised by the clinicians in the RCT and these categories have been used in this summary. The difference in percentage is provided along with the respective 95% confidence interval as this was the most consistently reported metric across all of the outcomes in the summary table (Table 6). The table shows the seizure remission rate has a 5% point benefit for zonisamide; however, the confidence interval is fairly wide and includes zero. The rates of adverse events are all higher in the zonisamide arm with differences of up to 9% where the confidence interval is much narrower and does not include zero in many cases.

**Table 6:** BRAT summary table for the SANAD II RCT

		**Outcome**	**Lamotrigine patients (*n* = 328)**	**Zonisamide patients (*n* = 324)**	**Difference (Z-L) in percentage (95% CI)**
**Benefits**	↑ Seizure remission	12-month seizure remission (5-year probability)	282 (86%)	295 (91%)	5 (− 5 to 16)
**Risks**	↑ Adverse events	Psychiatric disorders	43 (13%)	73 (23%)	9.4 (3.6 to 15)
		Nervous system disorders	53 (16%)	60 (19%)	2.4 (− 3.5 to 8.2)
		General disorders and administration site conditions	17 (5%)	39 (12%)	6.9 (2.6 to 11)
		Gastrointestinal disorders	25 (8%)	26 (8%)	0.4 (− 3.7 to 4.5)
		Skin and subcutaneous tissue disorders	24 (7%)	21 (7%)	− 0.8 (− 4.7 to 3.1)
		Investigations	6 (2%)	16 (5%)	3.1 (0.3 to 5.9)
		Metabolism and nutrition disorders	3 (1%)	16 (5%)	4 (1.4 to 6.6)
		Musculoskeletal and connective tissue disorders	5 (2%)	7 (2%)	0.6 (− 1.4 to 2.7)

##### Net clinical benefit

The net clinical benefit for zonisamide relative to lamotrigine is derived from the probability differences shown in the final column of Table 6. Using the formula given earlier, expected NCB is calculated as a weighted sum of the probability differences for benefits minus a weighted sum of the probability differences for risks, where the weights reflect the relative clinical importance of each outcome. Assuming for simplicity that all benefits and risks are weighted equally allows us to omit the weights from the formula and thus calculate the expected NCB for zonisamide relative to lamotrigine as:

$$\text{Expected NCB }= 5{\%}-\left(9.4{\%}+2.4{\%}+6.9{\%}+0.4{\%}-0.8{\%}+3.1{\%}+4{\%}+0.6{\%}\right)$$

$$\text{Expected NCB }= - 21{\%}$$

Since the expected NCB is negative, we conclude that on average lamotrigine has a more favourable benefit-risk balance than zonisamide, under the assumption that all benefits and risks are weighted equally. Should individual weights want to be included within the calculation of the NCB, further data would need to be collected from key stakeholders. An example can be found by Holmes et al. [41] where a discrete choice experiment was completed to estimate patient preferences in a related trial.

## Discussion

Using benefit-risk methods within these two case studies has shown a clearer presentation of the adverse event data collected within the RCT and provided these results with a more prominent reporting role alongside the key efficacy/effectiveness results. In both case studies, subjective clinical conclusions had been made about the treatments which considered both the effectiveness outcome/s and the adverse events. In particular, in the SANAD II case study, despite being found to be non-inferior, the authors did not recommend the test treatment as it also came with increased adverse events. It was shown how benefit-risk methods could potentially provide additional insights into this decision by summarising all relevant information together for the reader to interpret.

The format of each method for summarising the data differs with both the BRAT value tree and summary table providing clarity by collating all the necessary information into one place. The use of a summary table was one of the core recommendations from Cornelius et al. [4] to improve the reporting of adverse events and so using this method can satisfy this recommendation. The summary table also conforms to another of their recommendations which is to provide a summary of selected appropriate harms and include the information in the main body of the report as well as an interpretation of these results.

The OMERACT 3 × 3 table shows how the use of patient-level summaries of adverse events with efficacy/effectiveness outcomes can add to the conclusions of the study. Using the three different tables produced in Table 4, three different conclusions would be made about how the benefits and the risks of the treatment are related (if at all). Presenting this information adds further context to the results of the RCT and is important for clinicians to be able to appropriately assess the treatments.

The most simplified version of the net clinical benefit was implemented into the SANAD II case study. This put in numerical values the trade-off between the key benefit and the key risks which supported the clinical judgement made by the team. Using weighting within the NCB would be preferable in many cases if the outcomes are not deemed to be of equal importance; however, this would require additional data collection.

We believe that the use of these B-R methods has enhanced the reporting of the RCT results and allowed information to be presented to the reader in a useful and understandable format. However, each method has its strengths and weaknesses so the most useful methods for each case study may differ and should be decided on a case-by-case basis. The examples used in this article are not intended to be exhaustive and some of the more straightforward methods have been applied. Extensions to this work by using more in-depth methods, for example, by adding preference weightings to the outcomes, could be implemented to provide further context for any clinical decisions. The use of either qualitative or quantitative B-R methods is often debated within the literature [15]. Most guidance recommends solely qualitative methods (such as the BRAT framework) [42], but this is often criticised due to a lack of formal integration or consideration of preferences [15]. However, quantitative methods which are more computationally complex, negating some of the transparency which B-R methods aim to create [43]. The FDA [42] states that the use of quantitative B-R methods masks the nuance which is required for medical decision-making hence the primary recommendation of qualitative methods. For researchers using B-R methods to report RCTs, the appropriate level of complexity should be considered depending on the situation, requirements and stakeholders of interest.

Another consideration for researchers should be the level of harms used within the B-R method. The choice of this can vary depending on the method with some such as the OMERACT table requiring a three-group categorisation whereas other methods such as NCB using the adverse events individually within the method. As we recommend these methods to be used in conjunction with the CONSORT harms reporting standards [9], the decision of appropriate aggregated level of harm presentation within the B-R method can be selected on a case-by-case basis depending on the appropriateness of grouping harms.

The SANAD II case study is a non-inferiority trial where originally the main benefit was not expected to be better. This is the key design feature of a non-inferiority trial where the secondary outcome, often adverse events [44], is the outcome expected to be improved by the treatment. This potentially creates more need for the use of benefit-risk methods to summarise both the efficacy/effectiveness outcome and the harms when these are both important for the success of a treatment. In a non-inferiority investigation, there could be studies where there is a potential trade-off of a reduction in efficacy/effectiveness against added benefit in another area, i.e. an adverse event/s [45, 46]. Therefore, presenting the adverse events alongside the primary outcome becomes more important as without this information the investigative treatment may appear worse when considered in isolation of the harms.

## Conclusion

Adverse events suffer from poor reporting within RCTs, using benefit-risk methods can increase the prominence of these results by ensuring they are read alongside the primary efficacy/effectiveness outcomes. This ensures that all the factors which would be used to determine whether a treatment would be recommended or not are transparent to the reader.

## Data Availability

Data sharing is not applicable to this article as no datasets were generated or analysed during the current study.

## Abbreviations

B-R: Benefit-risk
BRAT: Benefit-Risk Action Team
CONSORT: CONsolidated Standards Of Reporting Trials
MedDRA: Medical Dictionary for Regulatory Activities
NCB: Net clinical benefit
RCT: Randomised controlled trial
OMERACT: Outcome Measures in Rheumatology
